# Mining expressed sequence tags identifies cancer markers of clinical interest

**DOI:** 10.1186/1471-2105-7-481

**Published:** 2006-11-01

**Authors:** Fabien Campagne, Lucy Skrabanek

**Affiliations:** 1 Institute for Computational Biomedicine and Dept. of Physiology and Biophysics, Weill Medical College of Cornell University; 1300 York Ave; New York, NY 10021, USA

## Abstract

**Background:**

Gene expression data are a rich source of information about the transcriptional dis-regulation of genes in cancer. Genes that display differential regulation in cancer are a subtype of cancer biomarkers.

**Results:**

We present an approach to mine expressed sequence tags to discover cancer biomarkers. A false discovery rate analysis suggests that the approach generates less than 22% false discoveries when applied to combined human and mouse whole genome screens. With this approach, we identify the 200 genes most consistently differentially expressed in cancer (called HM200) and proceed to characterize these genes. When used for prediction in a variety of cancer classification tasks (in 24 independent cancer microarray datasets, 59 classifications total), we show that HM200 and the shorter gene list HM100 are very competitive cancer biomarker sets. Indeed, when compared to 13 published cancer marker gene lists, HM200 achieves the best or second best classification performance in 79% of the classifications considered.

**Conclusion:**

These results indicate the existence of at least one general cancer marker set whose predictive value spans several tumor types and classification types. Our comparison with other marker gene lists shows that HM200 markers are mostly novel cancer markers. We also identify the previously published Pomeroy-400 list as another general cancer marker set. Strikingly, Pomeroy-400 has 27 genes in common with HM200. Our data suggest that a core set of genes are responsive to the deregulation of pathways involved in tumorigenesis in a variety of tumor types and that these genes could serve as transcriptional cancer markers in applications of clinical interest. Finally, our study suggests new strategies to select and evaluate cancer biomarkers in microarray studies.

## Background

Endogenous biomarkers are molecules whose levels in a tissue or fluid of an organism correlate with the presence of a given disease. For instance, the Prostate Specific Antigen (PSA) is an FDA-approved endogenous biomarker for prostate cancer [1,2]. Identifying biomarkers for specific cancer types is expected to lead to the development of early diagnostic methods that can reduce morbidity and mortality [3,4]. Here, we show that cancer biomarkers can be discovered by mining human and mouse Expressed Sequence Tags (ESTs). Furthermore, we show that biomarkers identified in this way perform well over the wide range of tumor types and classifications used in microarray studies. Our results demonstrate the existence of a small group of genes whose expression levels are affected in a wide array of cancers.

Various approaches have been used to search for cancer biomarkers. Methods that mine gene expression include Serial Analysis of Gene Expression (SAGE) [5,6], microarray analysis involving the comparison of tumor samples versus normal tissues (e.g., [7-10]), large scale meta analysis of cancer microarray data [11], or Massively Parallel Signature Sequencing (MPSS) [12]. Expressed Sequence Tags such as found in dbEST [13] are another abundant source of gene expression data. We have previously described TissueInfo, an approach to determine whole genome tissue expression profiles using data in dbEST [14]. In this article, we asked whether EST data could be mined to identify cancer biomarkers.

## Results and discussion

### Mining dbEST

**Figure 1:**
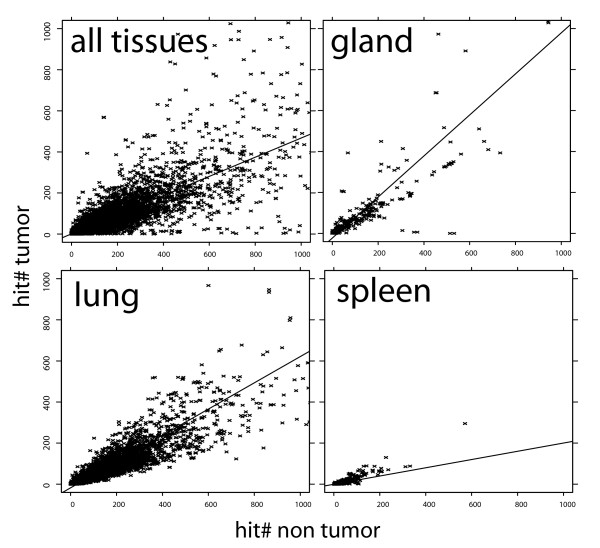
**Number of hits in non tumor tissues versus hits in tumor tissues**. The left top panel plots one point per transcript in the human genome (total number of hits <=1,000). The other panels were filtered to show only those transcripts for which the '*most expressed in*' TissueInfo attribute had value "gland", "lung", and "spleen". Filtering shows that transcripts *most expressed in *"gland" are on average equally likely to appear in tumor tissues as in non tumor tissues. In contrast, transcripts *most expressed in *"spleen" are five times more likely to appear in non tumor tissues. Filtering on other tissues shows average ratios of expression intermediate between gland and spleen. These plots reflect that EST libraries sequenced from different tissues have varied ratios of tumor/non tumor representation and that this ratio can be determined when grouping transcripts by the calculated *most expressed in *TissueInfo attribute. The consequence of grouping transcripts by the *most expressed in *attribute is that the scatter observed on the "all tissues" panel is greatly reduced after filtering. This observation motivated the development of the EST mining approach described in this manuscript.

We extended TissueInfo to mine ESTs for genes that are differentially expressed in non-tumor versus tumor tissues (see [14] for details about TissueInfo and Methods for the description of the cancer discovery extension). Figure 1 presents how many times human transcripts appear in tumor versus non-tumor tissues. The slope of the line that best fit the points is a consequence of how many EST libraries are available in tumor and non-tumor samples. The variation around the regression line, however, reflects both the random sampling effect of EST sequencing (sequencing effectively randomly picks mRNA molecules from the pool available in a given tissue sample) and the differential regulation of the genes between the tumor and non tumor conditions. The scatter observed when considering all transcripts (top left panel of Figure 1) can be considerably reduced when focusing on transcripts preferentially expressed in a given tissue. This noise reduction technique is central to the EST mining approach presented in this manuscript. After filtering, we used a two-tailed Fisher Exact Test to assign a P-value to each transcript and quantify the likelihood of differential expression between tumor and non-tumor (see Methods for details).

### Human-mouse comparison

We mined mouse ESTs in the same way and compared the results to those obtained with the human data. Ortholog information was obtained from Ensembl (see Methods for build numbers). Plotting the P-values obtained for human and mouse orthologs, we confirmed that the TissueInfo cancer P-value is uniformly distributed in regions where the P-value is not significant (e.g., 0.9 < P-value < 1, data not shown). Further, we asked if selecting genes in human by P-value provided more overlap with mouse than would be expected by chance if P-values were distributed randomly in the human/mouse ortholog gene set. We selected 355 human genes (P-value <= 1 10^-17^) and 186 mouse genes (P-value <= 1 10^-14^) and observed an overlap of 39 genes. Randomly selecting 355 genes and 186 genes in the human/mouse ortholog gene set yielded overlaps of ~3 (mean 3.25, max = 4, n = 4). The difference is significant (P < 1 10^-8 ^Fisher Exact Test using the maximum value, one tailed), indicating that the overlap observed cannot be explained by chance alone. These data are a strong indication that our approach works consistently in the two organisms, despite important differences in tissue and EST availability or cancer biology. The list of 39 genes is annotated and provided in Supplementary Table 1 [see Additional file 2]. The references cited in the table show that most genes on this list have been studied and found to be differentially regulated in cancer. Interestingly, of the 39 genes that both our mouse and human screens detect at high stringency, there is only one gene in common with the Perou-Brown-Botstein proliferation cluster [15]. This indicates that these 39 genes common between human and mouse are not the usual markers of proliferation and poor differentiation.

### False discovery rate

**Figure 2:**
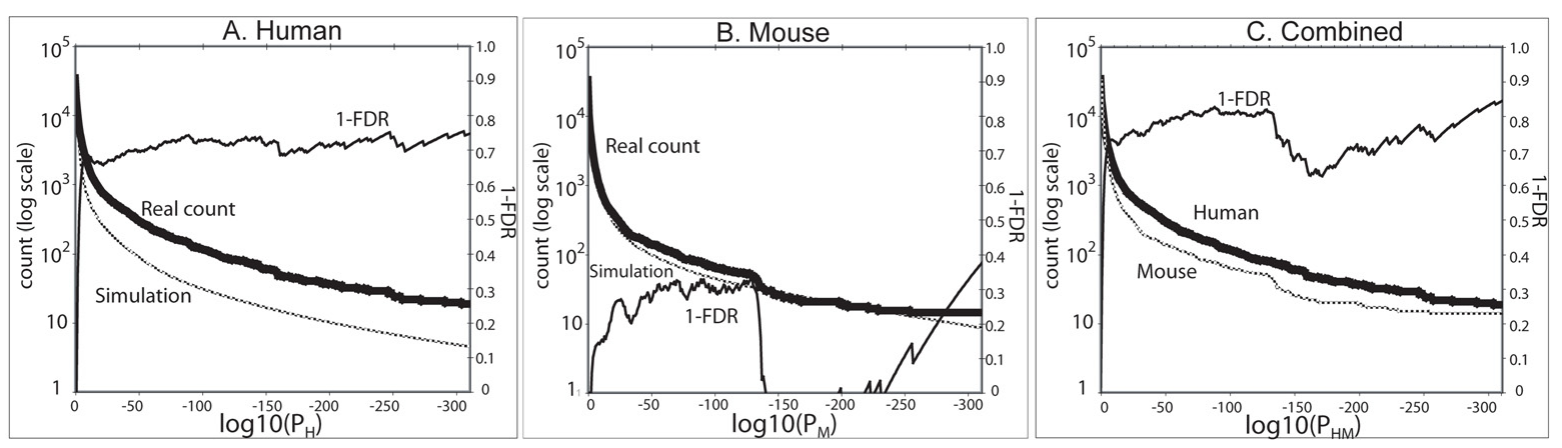
**False Discovery Rate Analysis**. A comparison of the number of transcripts (left axis) with differential expression found below various thresholds of the P-value in the data and in a control experiment for the human (Panel A) and mouse (Panel B) genomes. False Discovery Rate (see text) is shown on the right axis as 1-FDR. Panel C plots these values for the combined screen in which we combined P-values for the human and mouse experiment.

To confirm that small P-values indicate transcripts that are differentially expressed between tumor and non-tumor tissues, we estimated the False Discovery Rate, a statistical measure used to estimate the bias introduced by multiple hypothesis testing [16]. The False Discovery Rate (*FDR*) is the ratio *FDR *= *FP*/(*TP *+ *FP*), where *FP *is the number of false positives detected below a threshold and *TP *is the number of true positives (true positives are transcripts/genes that are differentially expressed between tumors and non-tumors and are selected by the method). To calculate *FDR*, *FP *and *TP *need to be known. We estimated *FP *as the number of genes selected in a control experiment (normal tissue vs. normal tissue). We estimated *TP *by comparing the control to the tumor vs. non-tumor data (*TP *= *R *- *FP*, where *R *stands for the number of genes selected in the tumor vs. non-tumor experiment). Figure 2 compares the number of transcripts found below various thresholds of the P-value in the data and in the control experiment, and plots 1-*FDR *as a function of the exponent of the P-value threshold. Figure 2 indicates that combining P-values for the human and mouse screens lowers the false discovery rate of our approach, from about 30% with the human data only to about 20% when human and mouse data are combined.

### HM200

We focused on human/mouse orthologous genes and selected the 200 genes that were ranked best by the combined human/mouse P-value P_HM_, where P_HM _= P_H _× P_M _(threshold P_HM_<= 1 10^-42.23^). We called this gene set HM200. Each gene which is part of HM200 has an orthologous counterpart in human or mouse, and has been selected because of differential expression in human cancer, mouse cancer, or both, as measured by our approach. From Figure 2 (panel C), we estimate that over 78% of the genes in HM200 are truly differentially expressed between tumor and non-tumor tissues. In contrast, if one were to randomly select genes from the genome to identify 200 genes truly significantly differentially expressed between tumor and non-tumors, the expected precision of the task would be 0.6015% (200/33,248). Using our approach with the combined human and mouse dbEST data is therefore 129 times (78%/0.6015%) more precise than a random selection.

### HM200 is enriched in cancer-associated genes

We asked if genes in HM200 were more likely to be oncogenes or tumor suppressor genes. To address this question, we counted how many genes in HM200 overlap with a set of 1,485 genes previously studied for their role in oncogenesis (the construction of this list was described in [17], see also Methods). We found that 16.5% of genes in HM200 overlap with this list (33/200), a number to be compared to 8.7% (1,403/16,093) in the whole human/mouse ortholog gene set. The difference is significant (P-value = 0.0011 Fisher Exact Test, two tailed), and comparable to the enrichment recently reported by Aouacheria and colleagues when identifying genes bearing cancer-associated nonsynonymous SNPs in dbEST [17].

### HM200 as predictive biomarkers

Although HM200 genes were identified in EST data, we reasoned that they should be accurate predictors in cancer microarray data sets. Testing HM200 on microarray data is an independent test of their biomarker abilities. This is a strong test because the only common point between microarray and expressed sequence tags is that these technologies measure gene expression. Therefore, to determine whether HM200 genes could be used as cancer biomarkers in human, we asked how well HM200 compared to other gene lists in a variety of microarray cancer classification tasks. Lists of gene markers have been derived in many microarray studies for different types of cancer and we collected 13 such gene lists from published studies (see Methods). Because gene lists vary in the number of genes that they contain, in addition to the HM200 gene list, we evaluated HM100, HM50 and HM10 gene lists with the top 100, 50 and 10 genes from HM200, respectively (see Methods). We also used a negative control gene list NC01-2000, of 420 genes with high TissueInfo cancer P-values (not expected to be accurate predictors). Although the complete list of genes on an array is not a practical biomarker list (some arrays contain more than 20,000 probes), we included the full set of genes found on the array used for each study as a positive control. In total, our evaluation compared the predictive performance of 19 gene lists over 24 different microarray datasets (listed in Supplementary Table 2 [see Additional file 3]). We included in this study all the microarray datasets that we could obtain. The number of gene lists included in the study was limited only by the computational cost of performing the evaluation (gene lists were not selected in any other way).

**Figure 3:**
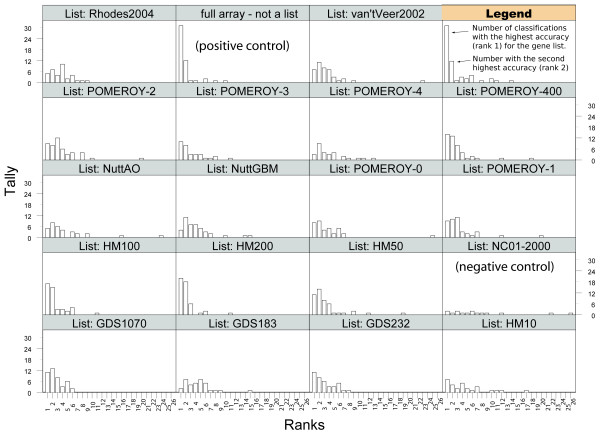
**Relative performance of gene lists compared across 1,121 classifications**. The number of classifications for which a list obtains a given accuracy rank is shown. Distributions skewed towards best ranks indicate gene lists that generalize well across different types of tumor classifications.

Microarray studies of cancer frequently compare two different conditions. In some studies, one condition is a tumor and the other the normal tissue. In other studies, conditions are derived from cytology, and tumor grades are used as conditions. The most interesting studies define conditions from data obtained about the patient after the tumor sample has been taken (these studies can be used to try and predict cancer outcome or response to treatment). The microarray datasets collected for this study include at least one representative of each of these types of classifications. Some microarray datasets are annotated such that multiple different classification tasks can be performed with them. For instance, in the Pomeroy2002 dataset, five classifications can be performed: normal cerebellum/medulloblastoma, normal/malignant glioma, normal/atypical teratoid or rhabdoid tumor, normal/primitive neuroectodermal tumor, and medulloblastoma/malignant glioma. In such cases, we included each possible classification if this classification had at least three patients in each class. Supplementary Table 3 lists the classifications considered in this study [see Additional file 4]. In our evaluation, the number of gene lists, datasets and classification conditions combine to produce 1,121 different cancer classification tasks. We evaluate the leave-one-out accuracy of each gene list on each classification task, filter out classification tasks for which the P-value of the label permutation test exceeds 0.05 (5% confidence level), and then rank gene lists in each dataset from highest to lowest accuracy (see Methods). Briefly, a gene list obtains rank 1 on a classification task if it yielded the highest accuracy for that task among the gene lists included in the evaluation. Figure 3 presents a tally of the rank for each gene list in our evaluation. (The data, programs and source code needed to reproduce the complete microarray evaluation are distributed as supplementary material and can also be downloaded from [18]). Supplementary Table 4 shows the statistical data used to build Figure 3 [see Additional file 5].

The 1,121 classification tasks measure two properties of each gene list: robustness and generality. We define robustness as the property of a biomarker set to perform well on independent datasets obtained for the same tumor type. Robustness should not be confused with leave-one-out performance, which estimates how well a predictor can generalize using information from a single training set. Robustness is a more important property than leave-one-out performance for clinical applications because any training set is unlikely to sample most of the variability found in the patient population. Thus, a good performance with one training set may not translate to another training set. For example, when we classify the Pomeroy2002 dataset using the GDS232 gene list ([7]), we test the robustness of the GDS232 gene list because both datasets include data from patients with medulloblastoma, and patients for these studies were recruited independently. These tests are performed to measure robustness. In contrast to robustness, we define generality as the property of a biomarker set to perform well on a tissue, tumor type or classification task other than the one it was trained for.

A perfect biomarker set is one that would be both robust and general. In the test presented in Figure 3, such a biomarker set would appear with one peak only, at rank 1, and the height of the peak would coincide with the total number of classifications performed in the evaluation. In contrast, the peaks of sets of genes unrelated to cancer should be randomly distributed across ranks and show no preference for the better ranks. Our negative control gene list NC01-2000 (see Methods) shows precisely this behavior (also, the negative control survives the permutation P-value filter in only one or two classifications per rank). Gene lists obtained from published studies generally show a skewed distribution towards the best ranks (e.g., Pomeroy-400, Pomeroy-1, GDS1070, GDS232). We observe that HM200 and HM100 present the most skewed distributions of the cancer marker gene lists. Given the range of classification tasks, tissue and tumor types sampled in our evaluation, Figure 3 indicates that the HM200 and HM100 marker sets are both robust and general cancer marker sets over the range of cancer classifications evaluated.

Prior comparisons of EST data and microarray data have confirmed that these two approaches to measuring gene expression have different strengths and drawbacks (including biases [19]). It is therefore notable to see that both EST and microarray data indicate that the genes in the HM200 gene list are differentially expressed in cancer and can predict cancer-related conditions (e.g., tumor/non-tumor, tumor cytology, likelihood of tumors to develop metastasis). This clearly shows that the HM200 gene list is not random and that our approach to mining expressed sequence tags is effective.

### HM200 are mostly novel biomarkers

We asked if genes in HM200 had been recognized before as biomarkers. We calculated the overlap between genes in HM200 and the 14 lists used in our evaluation (obtained from published studies, see Supplementary Table 5 [see Additional file 6], and the negative control). Numbers of genes overlapping between HM200 and these gene lists are shown in Table 1 with a gene list similarity measure (in the column labeled 'pseudo-p-value'). See Methods for calculations and a discussion of the validity of the pseudo P-values shown in Table 1. Table 1 shows that three of the previously reported cancer marker gene lists overlap more significantly with HM200 than others. These lists are Pomeroy-400-classic-desmoplastic (27 gene overlap), Pomeroy-class-1 (9 gene overlap), and GDS1070 (5 gene overlap). Interestingly, these three lists also showed better generality when tested across datasets than most other gene lists (excluding the HM gene lists) in the rank evaluation test shown in Figure 3. This shows that although 27 HM200 genes have been identified by Pomeroy and colleagues as central nervous system tumor markers, most genes in HM200 had not been previously recognized as cancer markers as a group. Table 1 also includes a comparison between HM200 and the proliferation cluster described by Perou, Brown, Botstein and colleagues in [15]. The comparison identifies only two genes in common between HM200 and the proliferation cluster The main contribution of our study is to show that a small group of genes (HM200 or subsets thereof) are predictive cancer markers that outperform other cancer marker lists over different tumor types and classification tasks. These results demonstrate the existence of a set of genes whose transcription levels are perturbed in a variety of tumors types, irrespective of the tissue of origin of the tumor.

**Table 1:** Gene overlap between HM200 and published cancer marker gene lists.

**List 1**	**List 2**	**Overlap**	**Size of list 1**	**Size of list 2**	**pseudo-p-value**
HM200	Pomeroy-400	27	200	305	1.04E-15
HM200	Pomeroy-1	9	200	103	5.41E-06
HM200	GDS1070	5	200	82	3.55E-03
HM200	NC01-2000	0	200	420	1.13E-02
HM200	GDS232	4	200	82	1.91E-02
HM200	NuttGBM	3	200	50	2.42E-02
HM200	vantVeer2002	4	200	93	2.87E-02
HM200	Pomeroy-3	4	200	95	3.07E-02
HM200	Perou-Brown-Botstein1999	2	200	31	5.65E-02
HM200	Pomeroy-2	4	200	132	8.25E-02
HM200	Rhodes2004	2	200	57	1.58E-01
HM200	GDS183	1	200	28	2.96E-01
HM200	Pomeroy-0	3	200	166	4.65E-01
HM200	NuttAO	1	200	76	6.14E-01
HM200	Pomeroy-4	0	200	92	6.33E-01

### HM200 and cancer pathways

Since the products of genes in HM200 are differentially expressed in tumor and non-tumor (as shown with EST data) and tumor grade or tumor evolution (as shown with microarray data), we hypothesized that HM200 would be likely to interact with genes shown to be altered in tumorigenesis (e.g., members of the central cancer pathways (reviewed in [3]). To test this hypothesis, we queried the Ingenuity Pathways Knowledge Base with accession codes for protein products of HM200. The Ingenuity Pathway database is a curated database that provides access to 1.3 million protein-protein and gene-protein interactions and offers tools that cluster genes into interaction networks. Supplementary Table 6 [see Additional file 7] and Supplementary Figures 1–10 [see Additional file 1] present gene networks identified when HM200 genes are used as focus genes (the best 10 networks, ranked by score are shown). Documentation available on the Ingenuity web site does not describe how the score is calculated, but indicates that the P-value of observing a network by chance is estimated to be 110^*-Score*^. The networks shown on Supplementary Table 6 [see Additional file 7] are thus unlikely to have been generated by chance (P-value≤1 10^-11^). Genes shown in bold (Supplementary Table 6 [see Additional file 7]) or in gray (Supplementary figures [see Additional file 1]) are members of HM200. Notably, network 1 contains the cancer-related genes FOS, FOSB, FOSL1, and JUNB; network 2 contains MYCN, BCL2, EGR2 [20]; network 3 contains Kinesin 5A, 5B, and 5C; network 4 contains MYC, network 5 contains the v-akt murine thymoma viral oncogene homolog 1 (AKT1/PKB); network 6 contains the retinoblastoma protein (RB1) and network 7 contains the P53 protein (TP53); network 10 contains the peroxisome proliferator-activated receptor γ (PPARG) and the granulocyte differentiation factor C/EBPα (CEBPA) [21]. Following this observation, we tested if genes found in these networks that are not HM200 biomarkers are enriched in cancer-related genes. Of the 195 genes in the 10 best networks shown in Supplementary Table 6 [see Additional file 7] that are not part of HM200, 27.69% (54/195) overlap with cancer-related genes (from reference [17]). In contrast, the cancer-related genes represent 10.11% (1,344/13,294) of human genes with a HUGO gene name (141 of the human genes have no HUGO identifier). The difference is significant (P-value 3.96 10^-9 ^Fisher Exact Test, two tailed). Interestingly, the overlap with cancer-related genes is significantly higher for genes known to interact with genes in HM200 than for genes in HM200 themselves (16.5%) (P < 0.01, Fisher Exact Text, one tailed). This supports our hypothesis that HM200 genes are interacting directly with genes involved in tumorigenesis and that their transcripts are markers of cancer-related signaling alterations. The cancer-related genes themselves may not be transcriptionally regulated, but expression of downstream genes such as those found in HM200 is.

### HM200 is enriched in mRNA binding proteins

We performed a Gene Ontology classification screen and found genes in HM200 to be significantly enriched in mRNA binding proteins (P-value 1 10^-4^, EASE score) [22]. This is not surprising because there is growing evidence that deregulation of protein translation may participate in malignant transformation [23,24]. The mechanisms by which tumor cells selectively deregulate protein synthesis are still unclear [25]. Therefore, the identification of genes that may participate in selective protein synthesis deregulation is of great interest. The networks provided as Supplementary Figures 1–10 [see Additional file 1] provide candidates for such genes. For instance, Supplementary Figure 3 [see Additional file 4] shows that seven genes in HM200 are connected to Jerky (JRK). The number of connections between JRK and HM200 genes is comparable to the number of connections between P53 and HM200. This is surprising because JRK was first studied as a temporal lobe epilepsy gene candidate [26].

More recent studies indicate that Jerky is an mRNA binding protein that binds mRNA through a CENP-B domain. Jerky shows selectivity in the set of mRNA molecules that it binds, and may regulate the availability of mRNAs to the translational machinery [27].

The CENP-B domain is found in nine other human proteins (CENP-B, peptidyl tRNA hydrolase 2 and seven Tigger transposable element proteins). The conservation of this domain suggests that some of these other proteins may also bind mRNA. However, the ability of these other proteins to bind mRNA selectively remains to be determined. We predict that Jerky (or one of the other nine CENP-B domain containing proteins) is involved in tumorigenesis by selectively deregulating protein translation. This hypothesis is supported by the presence of the Fragile Mental Retardation 1 protein (FMR1) on Network 8 (an mRNA binding protein connected to three HM200 genes) [27]. FMR1 binds mRNAs selectively and is believed to regulate their translation, thereby regulating the protein expression of specific genes [28]. FMR1 binds mRNAs through KH motifs. These motifs are also found in Nova-1, a biomarker for paraneoplastic opsoclonus-myoclonus ataxia disorder (such disorders affect the brain of some patients suffering from cancer and are not caused by metastasis) [29]. This discussion illustrates how interaction networks containing HM200 genes can be used to develop new molecular hypotheses for cancer biology.

### Potential issues when mining ESTs

We present and validate a new approach to identifying cancer biomarkers by mining expressed sequence tags. Because expressed sequence tags are a vastly heterogeneous type of data, any approaches that mine this source of data need to account for several issues. Many of these issues were discussed in our previous publication and will not be repeated here [14]. However, it is worth noting that the evaluation that we conducted in 2001 found that TissueInfo was accurate in identifying if a gene was expressed in a tissue in 80–89% of cases [14]. A new issue specific to mining ESTs for cancer markers is that dbEST contains different proportions of EST libraries from tumor and non-tumor tissues. This is clearly illustrated in Figure 1, panel "all tissues", where most transcripts are found to match double the ESTs in non-tumor tissues than in tumor tissues. In our new approach, we have addressed this issue in two ways. First, we identify groups of genes that are mostly expressed in a given tissue/organ or tissue type (e.g., gland). (We consider as many groups as there are tissues types annotated in the target organism.) Second, we calculate a Fisher Exact Test P-value which takes into account *(i) *the number of times a given transcript matches an EST in tumor, and in non-tumor, and *(ii) *the overall proportion of tumor/non-tumor ESTs for the tissue the transcript is mostly expressed in. This test adequately corrects for the tissues with varied proportions of ESTs in tumor and non-tumor tissues, and at the same time assigns significance as a function of the number of EST matches for the transcript. The second factor is important because we have shown that TissueInfo identifications are more accurate when a transcript matches more ESTs [14]. The False Discovery Rate analysis that we present in Figure 2 indicates that despite these various potential issues, our approach was able to identify genes differentially expressed in tumor/non-tumor tissues with about 80% precision (when the human and mouse genome screens are combined, Panel C). Additional independent validations, such as the overlap with cancer gene lists, the overlap between human and mouse predictions, and most importantly the large microarray evaluation study that we conducted, support these conclusions.

### Microarray validation confirms EST mining

The microarray evaluation study that we present in this manuscript is a large study by the current standards of the field. Indeed, this study considers a test set of 24 microarray datasets, when most marker discovery studies test a set of markers in one or two independently collected datasets. An exception is the study of Rhodes et al, where 12 datasets were used for independent validation [11] (The 40 datasets mentioned in the abstract of this publication were used as a training set and thus are not part of the independent test sets.) Furthermore, our validation study considers the set of markers that we identified in competition with 13 previously published genes lists, one negative control and a positive control (all genes on the array, see Figure 3 and Supp. Table 4 [see Additional file 5]). This contrasts with most published studies where single lists of markers are typically evaluated in isolation [7-9,11,12,30,31]. We used a stringent permutation test to evaluate the impact of the number of samples in each condition and intra-dataset correlations to the performance observed in a classification (this test, described in detail in [32] is stronger than the test used in [11]). The negative control gene list is not expected to perform at a statistically significant level and indeed, most of its classifications are filtered out by the permutation test (see Figure 3). Applying this stringent protocol to the comparison of multiple marker lists on different datasets and classification tasks revealed that some marker lists are more general than others. (We defined generality as the property of a marker list to be predictive on classification tasks and tumor types other than the one the markers were initially selected for.) To the best of our knowledge, the generality of cancer marker lists has not been studied before. HM200 achieves the best or second best classification in 79% of the classifications considered (i.e., those that survived the permutation test). Further, we report that one previously published list (Pomeroy-400 from [33]) also shows good generality across tumor types. Our observation that some marker lists are predictive over a wide range of tumor types and classification types strongly suggests the existence of a subset of genes under the transcriptional control of pathways consistently deregulated in different cancer types. (This interpretation is supported by the pathway analysis presented above and concurs with the view presented in [3]). Our results also suggest that new cancer marker studies should report on the generality of the markers that they identify because results obtained with general markers are more likely to be reproducible.

### HM200 applications

We anticipate that the discovery of the HM200 cancer marker gene set and the characterization of its predictive utility across tumor types will open new strategies for the identification of genes whose alterations are required steps in tumorigenesis (for instance, mining of microarray data for robust and general cancer marker genes can be envisioned). Also, since HM200 markers are predictive across tumor types, a subset of these markers can be selected *a priori *for gene selection in microarray data, and complemented with genes selected from the specific study. This strategy will substantially reduce the amount of over-learning that occurs when genes/features are selected from the same dataset that is used for classification. Further, patient diagnostics will benefit from the knowledge that a small set of genes are predictive for a large array of cancers, because molecular tools that are more sensitive and accurate than microarrays (e.g., antibodies, primers for RT-PCR, etc.) can be developed and used with small gene lists.

## Conclusion

We have presented a new approach to mining expressed sequence tags to discover cancer transcriptional markers. We validated this new approach with a series of tests, including False Discovery Rate analysis. We used this new approach to identify a set of 200 cancer biomarker genes (the HM200 biomarker set) from human and mouse EST data. We further validated HM200 with data from 24 publicly available microarray datasets and showed that, as a set, HM200 genes were effective in predicting conditions of clinical interest. The microarray validation protocol which we describe in this manuscript helps compare the predictive ability of a given gene list against previously published gene lists and helps assess the level of generality and robustness of the gene list under study across a variety of conditions. With this protocol, we showed that HM200 is a general cancer biomarker set which is predictive across a variety of tumor types and clinical conditions. The manuscript also presents a pathway analysis of genes in HM200 which suggests that genes in HM200 are under transcriptional control of pathways known to be deregulated in tumorigenesis.

## Methods

### Human and mouse transcripts data

We obtained known and predicted transcripts from Ensembl (human build NCBI35, mouse build NCBIM33). Ensembl transcripts were filtered for repetitive sequence regions with RepeatBeater (graciously provided by Dr. Coward). Similarity searches between ESTs and Ensembl transcripts were conducted for each organism (human or mouse) with megablast [34]. ESTs that matched transcripts with less than 95% sequence identity or over less than 150 base pairs were rejected (timegablast parameters – *error 0.05 -required-length 150 -assemble-hsps*).

### Mining of dbEST

Our EST mining pipeline is based on TissueInfo, previously described in [14]. Briefly, TissueInfo identifies ESTs that match a set of query sequences and provides curated data and analysis tools to query the set of matching ESTs for information about the tissues and organs from which the EST was sequenced. Curated data consists of a tissue ontology (formerly called TissueHierarchy). The TissueInfo ontology is provided in OWL format on the TissueInfo web site [35]. TissueInfo also provides tissue annotations that map most ESTs in dbEST to concepts in the TissueInfo ontology (formerly called curated tissue information). This mapping makes it possible to effectively mine data from multiple EST sequencing projects. Together, the ontology and the EST annotations provided by TissueInfo support the rich semantic queries needed to implement the cancer biomarker approach described here.

### Cancer TissueInfo extensions

To mine dbEST for transcripts differentially expressed in cancer, we extended TissueInfo as follows: (1) We extended EST annotations to note if the EST was sequenced from a tumor tissue. For instance, if an EST library description is *"cervical tumor"*, we annotate this EST with *"+cervix, /cancer"*, to reflect the fact that the tissue anatomical provenance was cervix, and that the tissue was tumorous. (2) We implemented ways to calculate hitTumor() and hitNonTumor() for each query sequence. The calculation hitTumor() returns the count of ESTs matching the query sequence whose tissue provenances have the /*cancer *attribute. Conversely, the calculation hitNonTumor() returns the count of ESTs that do not have the /*cancer *attribute. (3) We devised and implemented a P-value calculation method to allow ranking transcripts by the likelihood that they are differentially expressed in cancer tissues (see below).

### Cancer P-value calculations

As shown in Figure 1, grouping transcripts by the tissue in which they are most abundantly expressed (mostExpressedIn() calculation, see [14]) removes the variation of the ratio hitTumor()/hitNonTumor() observed across tissues. We define groups of transcripts *G *such that each transcript *t *in the group is mostly expressed in the same tissue (i.e., *t.mostExpressedIn*() is constant in a group *G*). For each transcript *t *in a group *G*, we define:

*x *= *t.hitTumor*()

*y *= *t.hitNonTumor*()

N1=∑Gx;N2=∑Gy
 MathType@MTEF@5@5@+=feaafiart1ev1aaatCvAUfKttLearuWrP9MDH5MBPbIqV92AaeXatLxBI9gBaebbnrfifHhDYfgasaacH8akY=wiFfYdH8Gipec8Eeeu0xXdbba9frFj0=OqFfea0dXdd9vqai=hGuQ8kuc9pgc9s8qqaq=dirpe0xb9q8qiLsFr0=vr0=vr0dc8meaabaqaciaacaGaaeqabaqabeGadaaakeaacqWGobGtdaWgaaWcbaGaeGymaedabeaakiabg2da9maaqafabaGaemiEaGhaleaacqWGhbWraeqaniabggHiLdGccqGG7aWocqWGobGtdaWgaaWcbaGaeGOmaidabeaakiabg2da9maaqafabaGaemyEaKhaleaacqWGhbWraeqaniabggHiLdaaaa@3DC0@

From these definitions, we can calculate the TissueInfo cancer P-value:

*P - value*_*cancer *_= *FisherTwoTail*(*x*, *y*, *N*_1_, *N*_2_)

where FisherTwoTail is a Fisher Exact Test (two tailed), as described and implemented in [36].

### False discovery rate

The control experiment is designed to estimate the number of transcripts that are falsely marked as being differentially expressed between tumor and non-tumor tissue at different thresholds of the P-value. Each query transcript matches a number of ESTs that can come from a number of different libraries. In the control experiment, all non-tumor libraries are randomly split into two groups based on their raw tissue information. For instance, the raw tissue labels "whole eye", "eye", "eye anterior segment", "Pterygium" or "Trabecular meshwork " are all equivalent to +eye in the TissueInfo ontology; each raw tissue designation is randomly assigned to one group or the other at each step of the simulation. (Because there are a number of raw tissue designation for a given tissue type, and because groupings are redone at each simulation step, the grouping procedure is not expected to result in more homogeneous groups in the control than in the real experiment.) After this grouping, the number of ESTs matching a transcript in each group is counted. The P-value of the difference of the counts in the two library sets is then calculated as for the cancer P-value calculations above, grouping transcripts by the tissue in which they are most abundantly expressed. This control experiment was repeated 10,000 times. We counted the number of transcripts identified below a certain P-value for a genome and averaged this number over the number of simulations. This value is the estimated number of false positives (FP) observed in the control, and can be used to calculate the False Discovery Rate (FDR) as shown in Figures 2A and 2B. Figure 2 indicates that when mouse and human data are combined, the approach presented in this manuscript generates less than 22% false positive predictions.

### Gene list constructions

Gene lists were obtained from the supplementary material of published articles whenever possible. We collected 13 gene lists from six microarray studies [7-9,30,33,37] and one meta analysis study [11]. In addition to HM200, we evaluated smaller subsets of HM200 constructed by selecting the 10, 50, and 100 genes ranked highest by the TissueInfo cancer P-value. We called these lists HM10, HM50, HM100. We constructed a gene list to be used as negative control (NC01-2000) by selecting 420 genes with a TissueInfo cancer P-value between 0.9 and 1.0. Genes in NC01-2000 are not expected to be good predictors in cancer classification tasks. Finally, for each dataset, we considered the gene list that consists of all the probesets on that array (gene list: full). Accession code conversions (e.g., from Affymetrix to Genbank, or from SwissProt to Ensembl gene IDs were done using Ensmart/Biomart [38]). The Rhodes2004 gene list was obtained by manually typing the HUGO IDs that appear in Figure 2 of [11]. The figure contains 67 gene names, but only 57 of these mapped to HUGO gene names and could be mapped to Ensembl gene IDs (some genes could not be identified unambiguously by the name listed in the Figure, for instance "p100" or "OK/SW-ol56"). Gene lists used for our evaluation can be found with the programs distributed as Supplementary Material to this manuscript.

### Microrray datasets

The datasets used in our evaluation were obtained from the Gene Expression Omnibus [39], the Broad Institute web site [40], or the Tmm database at Columbia University[41,42]. Datasets were parsed and processed with the TissueInfo software (distributed under the GPL and available from [35]). A complete list of datasets is given in Supplementary Table 2 [see Additional file 3].

### Microarray classification tasks

Supplementary Table 3 [see Additional file 4] lists the conditions and number of samples for each classification task for which a machine learning classifier was trained (see below).

### Machine learning

A Support Vector Machine (SVM) is a modern machine learning algorithm [43] that has been found to outperform other machine learning approaches (e.g., artificial neural networks) in a variety of application domains and applications [41,44]. Array signals were mean normalized across each condition of the array. After normalization, and following [33], low signals were set to a constant signal, Specifically, for one channel arrays, if the signal was lower than *f*_1_, the signal was set to *f*_1 _(we used *f*_1 _= 300 consistently throughout the evaluation). For two color arrays, if abs((*signal*-1)+1) <= *f*_2_, the signal was set to 1.0 (we used *f*_2 _= 1.15 consistently throughout the evaluation). This signal transformation sets low signals to a constant value. When used with feature scaling (see below), this significantly reduced the impact of noisy features on the outcome of the predictions. The resulting signals were used as features of the SVM. Features were scaled to the range [-1,+1] to reduce the risk that features with large variance dominate the decision function of the SVM. Learning was performed with a linear kernel. Since we trained with small training sets (n < 50), we did not optimize the learning parameter C of the SVM. This parameter was set consistently for each dataset and classification to the value *n*/x→
 MathType@MTEF@5@5@+=feaafiart1ev1aaatCvAUfKttLearuWrP9MDH5MBPbIqV92AaeXatLxBI9gBaebbnrfifHhDYfgasaacH8akY=wiFfYdH8Gipec8Eeeu0xXdbba9frFj0=OqFfea0dXdd9vqai=hGuQ8kuc9pgc9s8qqaq=dirpe0xb9q8qiLsFr0=vr0=vr0dc8meaabaqaciaacaGaaeqabaqabeGadaaakeaacuWG4baEgaWcaaaa@2E37@·x→
 MathType@MTEF@5@5@+=feaafiart1ev1aaatCvAUfKttLearuWrP9MDH5MBPbIqV92AaeXatLxBI9gBaebbnrfifHhDYfgasaacH8akY=wiFfYdH8Gipec8Eeeu0xXdbba9frFj0=OqFfea0dXdd9vqai=hGuQ8kuc9pgc9s8qqaq=dirpe0xb9q8qiLsFr0=vr0=vr0dc8meaabaqaciaacaGaaeqabaqabeGadaaakeaacuWG4baEgaWcaaaa@2E37@, where *n *is the number of training examples and x→
 MathType@MTEF@5@5@+=feaafiart1ev1aaatCvAUfKttLearuWrP9MDH5MBPbIqV92AaeXatLxBI9gBaebbnrfifHhDYfgasaacH8akY=wiFfYdH8Gipec8Eeeu0xXdbba9frFj0=OqFfea0dXdd9vqai=hGuQ8kuc9pgc9s8qqaq=dirpe0xb9q8qiLsFr0=vr0=vr0dc8meaabaqaciaacaGaaeqabaqabeGadaaakeaacuWG4baEgaWcaaaa@2E37@ is the feature vector. We measured the performance of the SVM with the leave-one-out protocol. This protocol provides a nearly unbiased estimate of the classification error and is tractable for small training sets [45]. We performed the machine learning evaluation with two different support vector machine implementations (Thorsten Joachims' SVMlight program [46] and libSVM). Results were qualitatively similar across implementations (data shown for the SVMlight evaluation).

### Classifier P-Values

Similar to [32], we estimate the significance of the classifiers trained from each dataset/classification task. This test evaluates the likelihood that the machine learning performance observed on the training set is the result of correlations in the features unrelated to the class that the classifier is trained to predict. For microarray data, features encode information about the expression levels of genes, which may contain an experimental noise/error component. Other factors that can affect classification tasks are the size of the training set and the number of features. In this test, many artificial training sets are constructed by shuffling the labels of the reference training set. This will not affect the size of the training set or the number of features, but will control for random noise. Each artificial training set is used to train and evaluate a classifier (see Machine Learning above). The test counts how many classifiers built with artificial training sets achieve the same performance as, or better than, the reference training set. Dividing this count by the number of artificial training sets tested yields a P-value that indicates how dependent the performance measured for the reference training set is on experimental noise or feature distributions. Classifier P-values reported in this manuscript were obtained after constructing 1,000 artificial training sets for each dataset/classification pair.

### Ranking gene lists

SVMs are trained for a classification task with different gene lists. This results in multiple accuracy measurements for each classification task (there is one accuracy for each gene list on a given classification task). To facilitate comparison of the predictive ability of gene lists, we rank gene lists by decreasing leave-one-out accuracy on each classification task. The gene list that obtains the best leave-one out accuracy is assigned a rank of 1 (if several gene lists reach the same accuracy, they are given equal rank). The gene list with the second best accuracy is assigned a rank of 2, and so on. Supplementary Table 4 [see Additional file 5] lists the raw performance measures and the ranks for each combination of gene list and classification task in our evaluation.

### Comparing gene lists

In Table 1, we use a Fisher Exact Test to determine which gene lists are more similar to HM200. The result of this test must be considered a pseudo P-value, because we do not control for correlations that may exist across the gene lists and would create artificially low P-values. A number of such correlations can be excluded because we always compare gene lists obtained from microarray and expressed sequence tags (excluding the comparison between HM200 and NC01-2000). For instance, this protocol excludes correlations due to the ability of probes to hybridize to the array (since this effect does not apply to HM200). However, we cannot totally exclude the possibility that the overlap observed in Table 1 is due to the level of expression that is necessary to detect gene expression with either microarray data or with our EST mining approach (this effect would reduce the total pool of genes that should be considered in the Fisher Exact Test). To acknowledge this effect, we report a pseudo P-value and use it as a gene list similarity measure. The pseudo P-value is useful because it takes into account the effect of different gene list sizes, while the number of genes that overlap do not.

### Description of ingenuity pathways analysis

Data in Supplementary Table 6 [see Additional file 7] were generated through the use of Ingenuity Pathways Analysis, a web-delivered application that enables biologists to discover, visualize and explore therapeutically relevant networks significant to their experimental results, such as gene expression array data sets. For a detailed description of Ingenuity Pathways Analysis, see [47].

Swiss-Prot accession codes for proteins coded by genes in the HM200 gene set were uploaded to Ingenuity Pathways Analysis. Each gene accession code was mapped to its corresponding gene object in the Ingenuity Pathways Knowledge Base. These genes, called Focus Genes, were then used as the starting point for generating biological networks. To start building networks, the application queries the Ingenuity Pathways Knowledge Base for interactions between Focus Genes and all other gene objects stored in the knowledge base, and generates a set of networks with a network size of 20 genes/proteins. Ingenuity Pathways Analysis then computes a score for each network according to the fit of the user's set of significant genes. The score is derived from a P-value and indicates the likelihood of the Focus Genes in a network being found together due to random chance. A score of 2 indicates that there is a 1 in 100 chance that the Focus Genes are together in a network due to random chance. Therefore, scores of 2 or higher have at least a 99% confidence of not being generated by random chance alone. Biological functions are then calculated and assigned to each network.

Biological functions were assigned to each gene network by using the findings that have been extracted from the scientific literature and stored in the Ingenuity Pathways Knowledge Base. The biological functions assigned to each network are ranked according to the significance of that biological function to the network. A Fisher Exact Test is used to calculate a P-value determining the probability that the biological function assigned to that network is explained by chance alone.

### GO category analysis

We searched GO functional categories for categories statistically enriched in HM200. We used the EASE software [22] with 189 SwissProt/UniProt identifiers (from HM200) and compared to 17,915 human proteins in UniProt. The P-value reported is the EASE Score [22].

## Authors' contributions

FC designed implemented and performed the EST mining approach, and most evaluation studies. LS annotated EST libraries, curated the TissueInfo ontology, performed the FDR simulations and collected microarray datasets. Both authors wrote the manuscript.

## Supplementary Material

Additional file 1 Description of supplementary files. Description of supplementary files and Supplementary Figures 1–10 (graphical view of Ingenuity Networks). Click here for file

Additional File 2 Supplementary Table 1. Description and annotation of 39 genes identified by both the human and the mouse dbEST screens. Click here for file

Additional file 3 Supplementary Table 2. Microarray evaluation datasets used. Click here for file

Additional file 4 Supplementary Table 3. Descriptions of the classifications tasks performed in the microarray evaluation study. Click here for file

Additional file 5 Supplementary Table 4. Evaluation statistics. Click here for file

Additional file 6 Supplementary Table 5. Description of the gene lists. Click here for file

Additional file 7 Supplementary Table 6. Ingenuity networks in tabular format. Click here for file
