# Supplementary material for: Mining expressed sequence tags identifies cancer markers of clinical interest
Source: BMC Bioinformatics. 2006 Nov 1;7:481. doi: 10.1186/1471-2105-7-481 (PMC1635568; doi:10.1186/1471-2105-7-481)
Supplement: Additional file 1 — Description of supplementary files. Description of supplementary files and Supplementary Figures 1–10 (graphical view of Ingenuity Networks). [file 1471-2105-7-481-S1.pdf]

Mining expressed sequence tags identifies cancer markers of clinical interest

Fabien Campagne* and Lucy Skrabanek

Institute for Computational Biomedicine and Dept. of Physiology and Biophysics, Weill Medical College of Cornell University; 1300 York Ave; New York, NY 10021, USA

* To whom correspondence should be addressed (fac2003@med.cornell.edu).

Supplementary Material

The following information is provided as supplementary material to the article:

Supplementary Table 1

Description and annotation of 39 genes identified by both the human and the mouse dbEST screens. (See Individual Excel file.)

Supplementary Tables 2-5

Details and results of the microarray evaluation study. (See Individual Excel files.)

Supplementary Table 6

Protein interaction networks in which HM200 markers participate. (See Individual Excel file.)

Supplementary Figures 1-10

Each network listed in Supplementary Table 1 is presented graphically. (See following pages.)

Additional supplementary material (source code, evaluation data sets and gene lists) are provided on the TissueInfo web site:
http://icb.med.cornell.edu/crt/tissueinfo/index.xml

Supp. Figure 1. Network 1

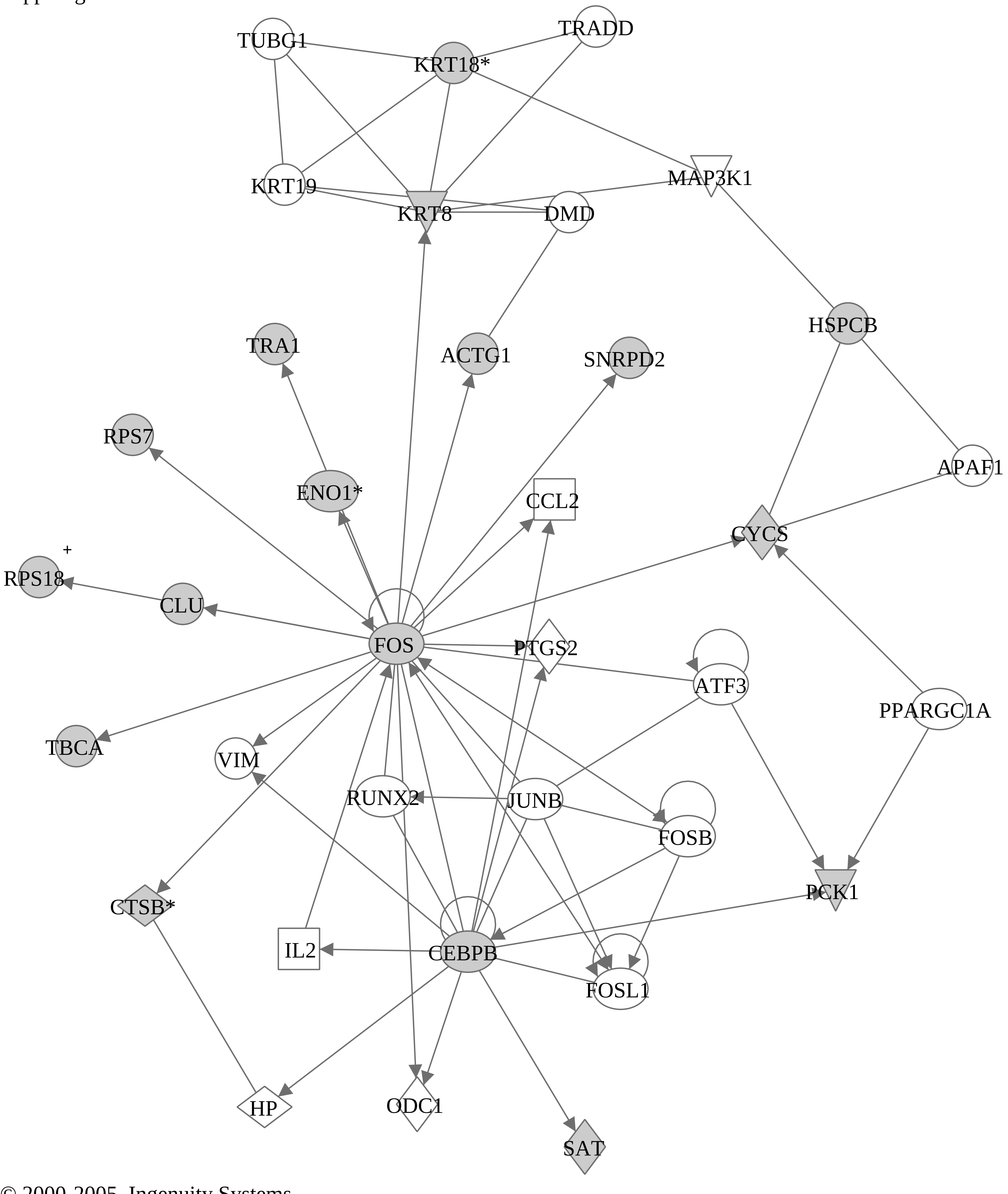

© 2000-2005, Ingenuity Systems

Supp. Figure 2. Network 2

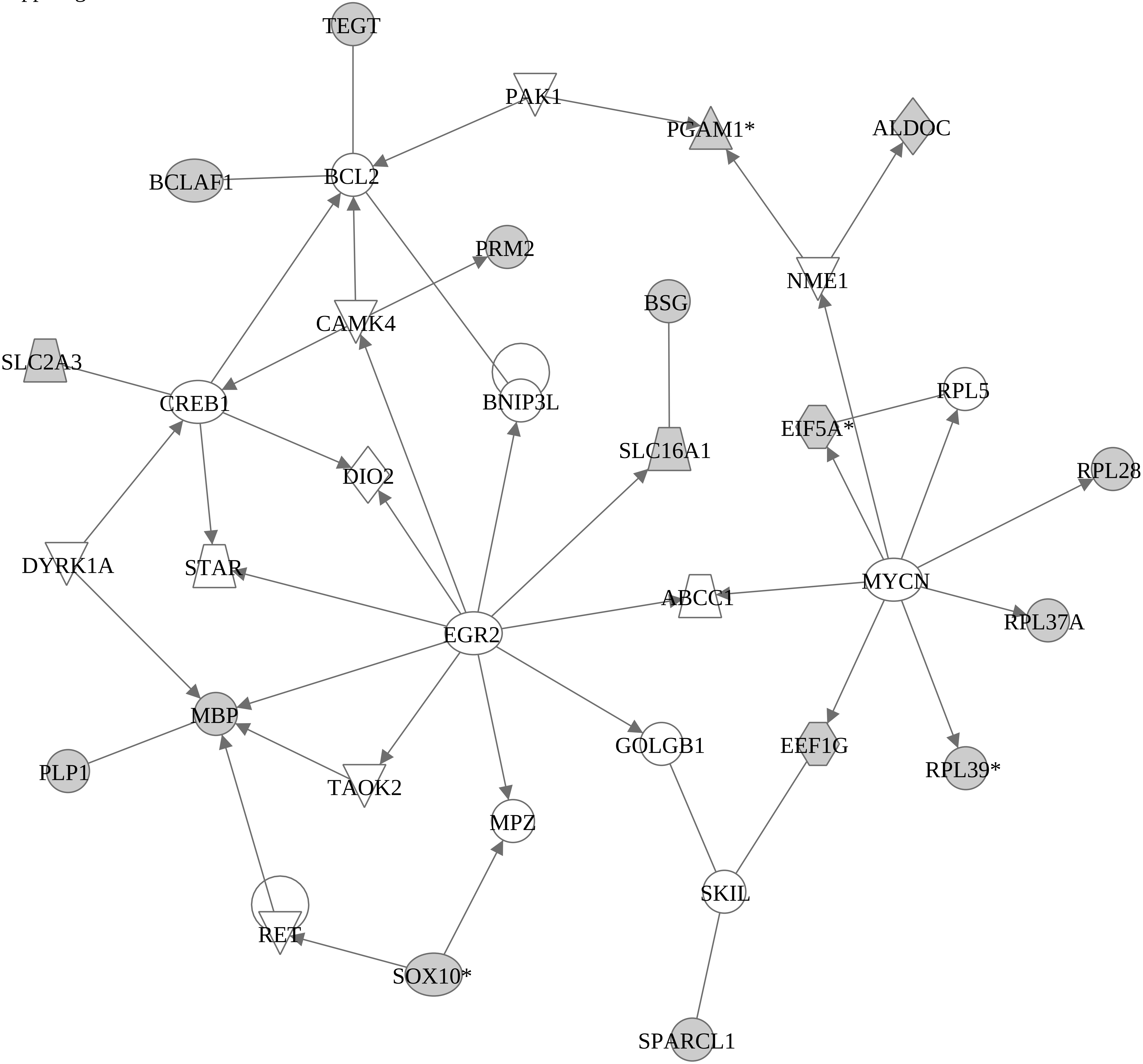

© 2000-2005, Ingenuity Systems

Supp. Figure 3. Network 3

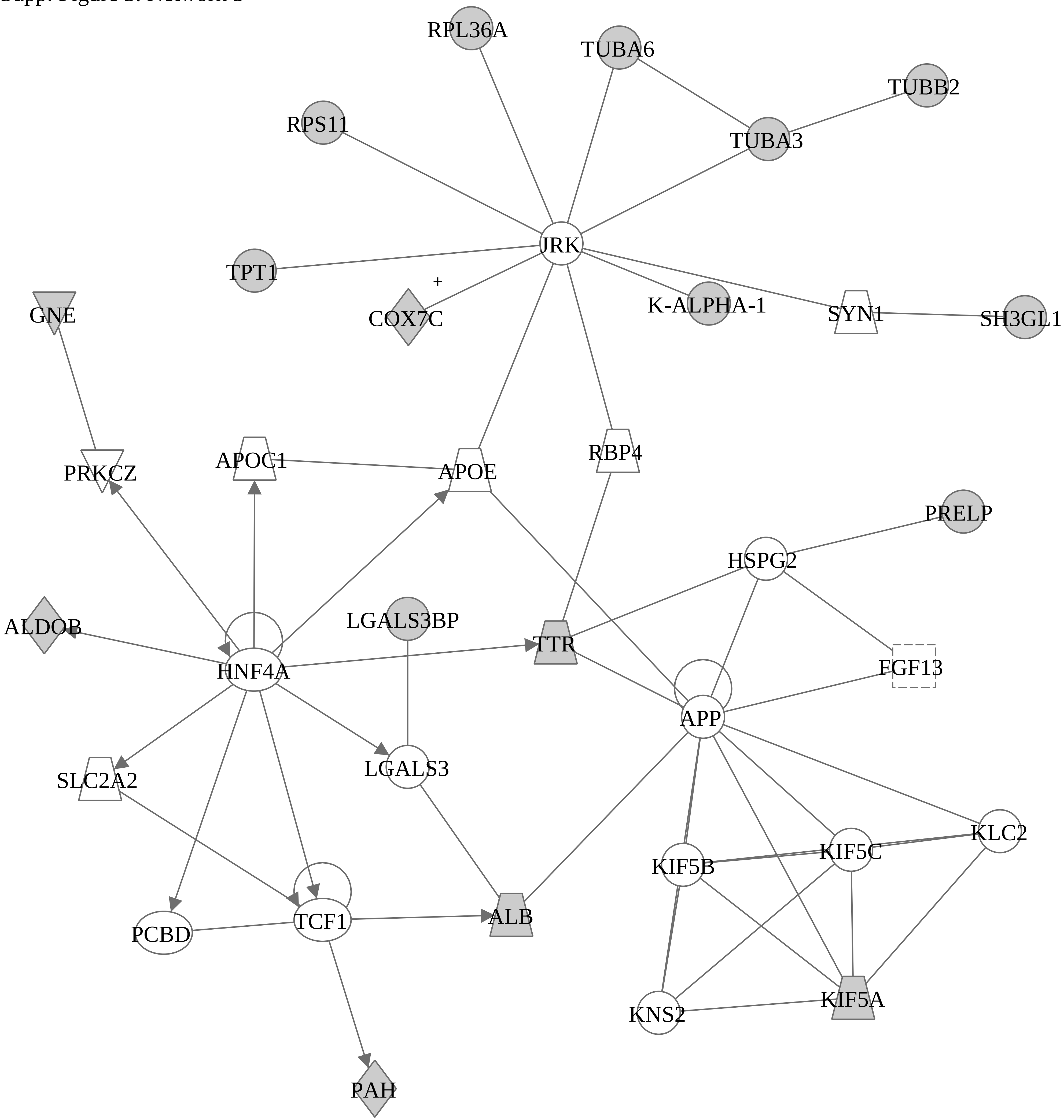

© 2000-2005, Ingenuity Systems

Supp. Figure 4. Network 4

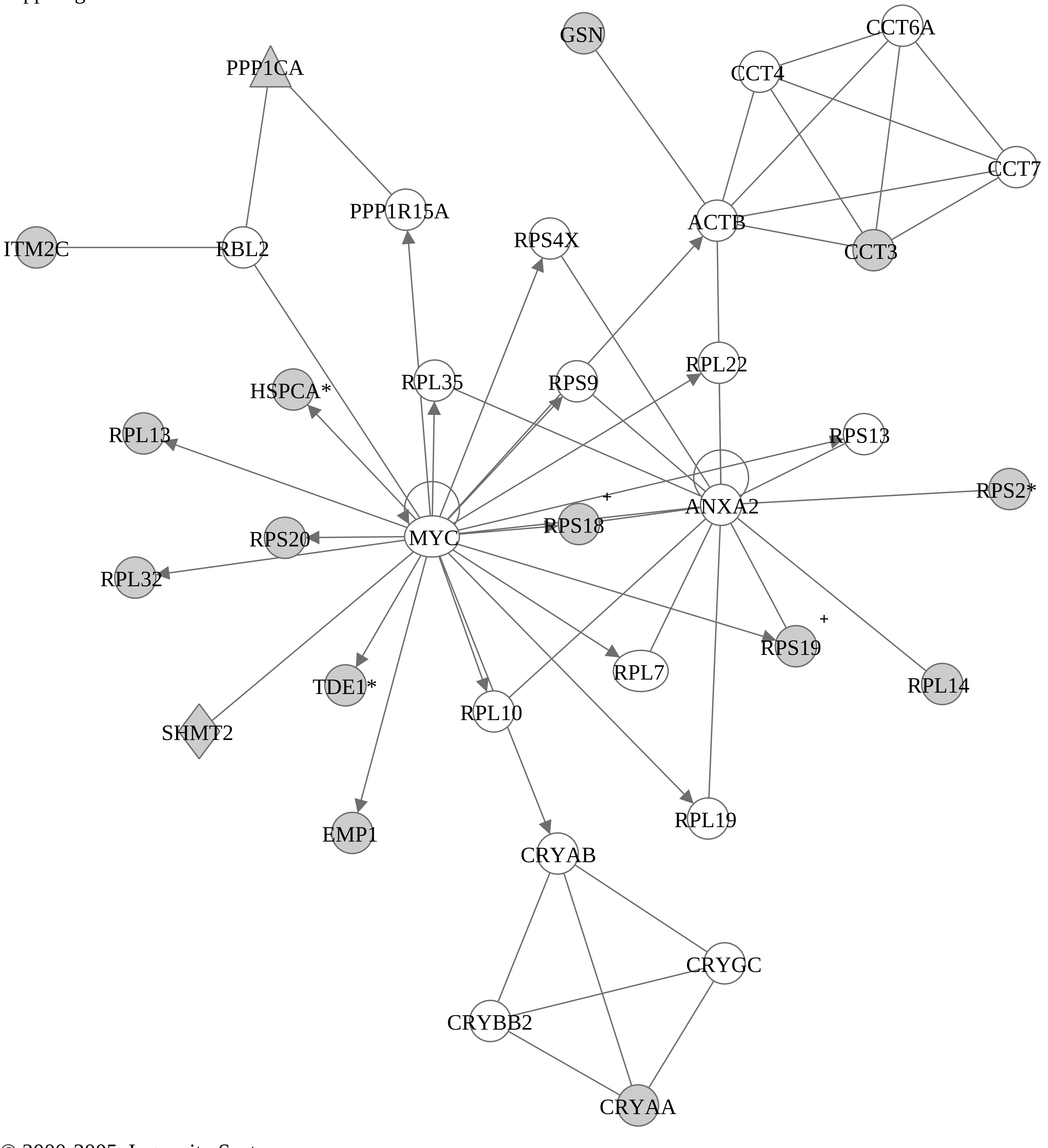

© 2000-2005, Ingenuity Systems

Supp. Figure 5. Network 5

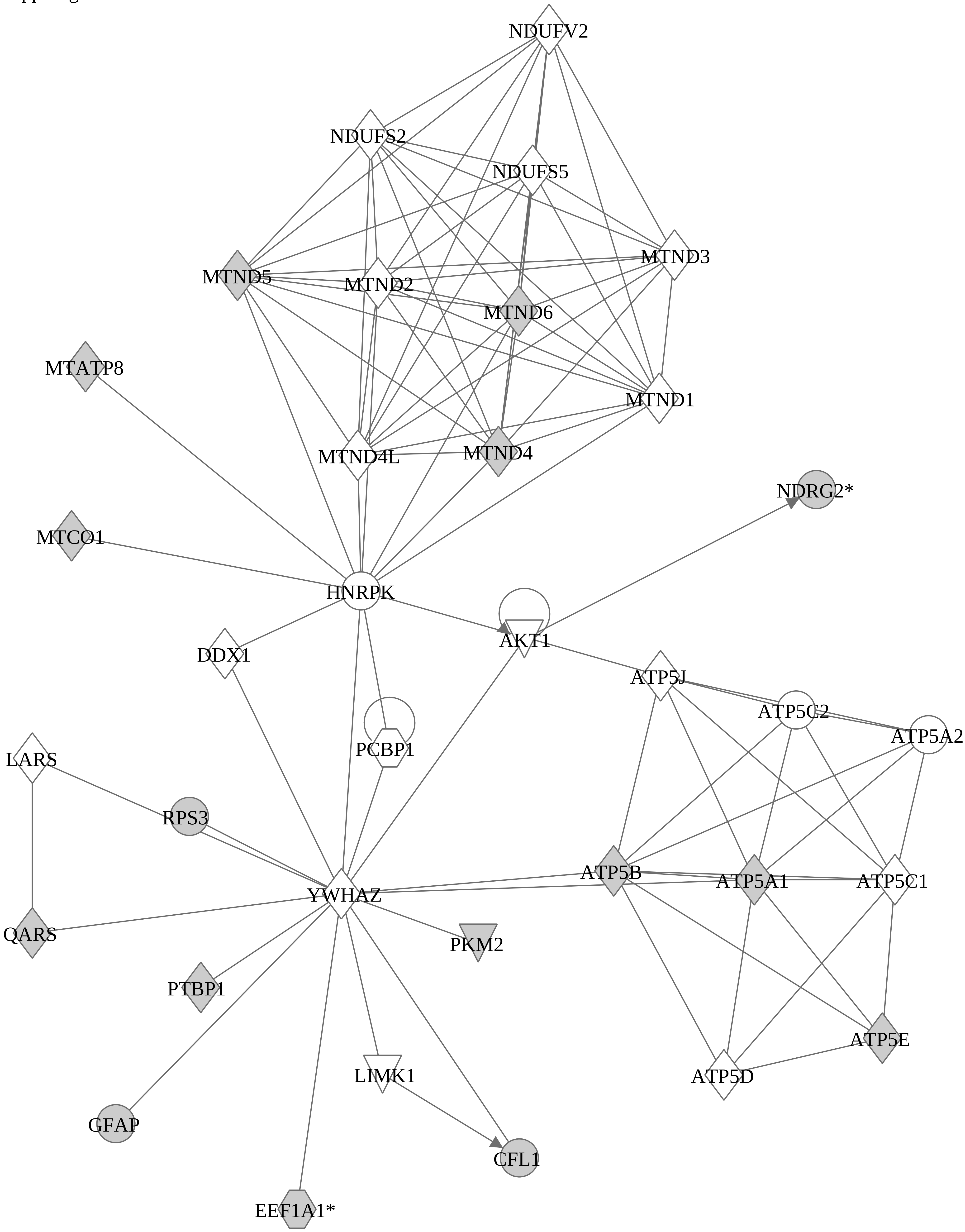

© 2000-2005, Ingenuity Systems

Supp. Figure 6. Network 6

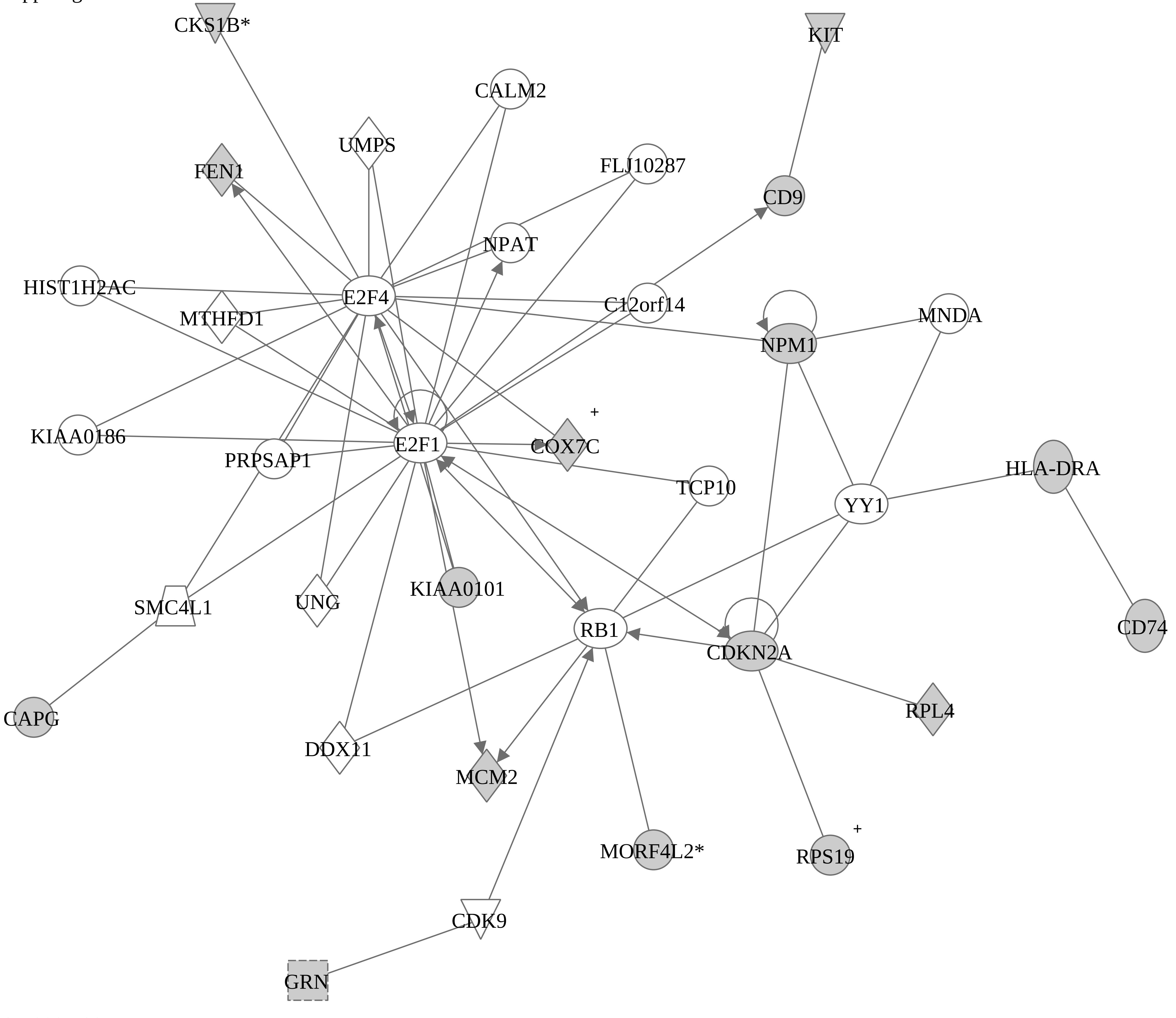

© 2000-2005, Ingenuity Systems

Supp. Figure 7. Network 7

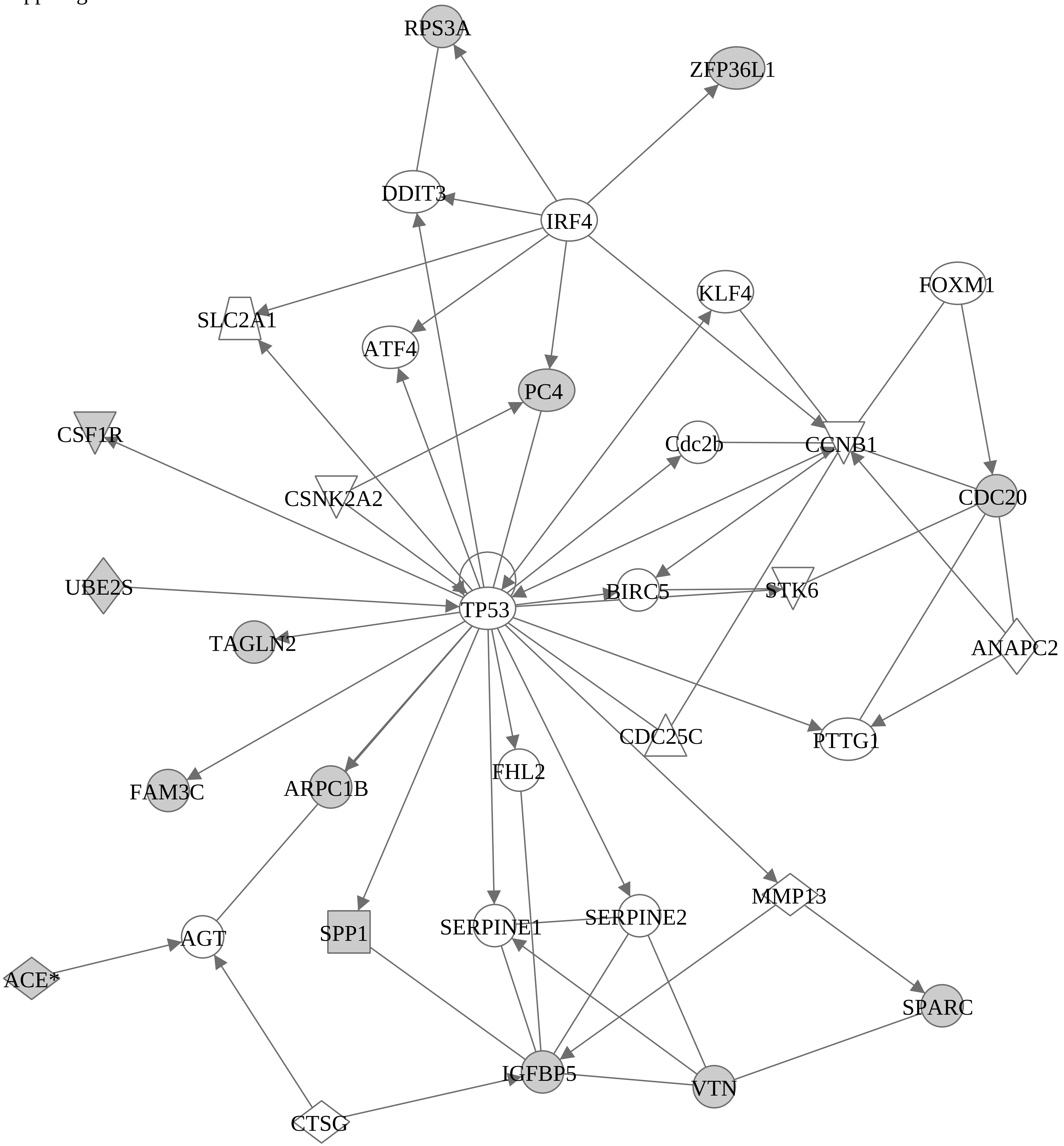

© 2000-2005, Ingenuity Systems

Supp. Figure 8. Network 8

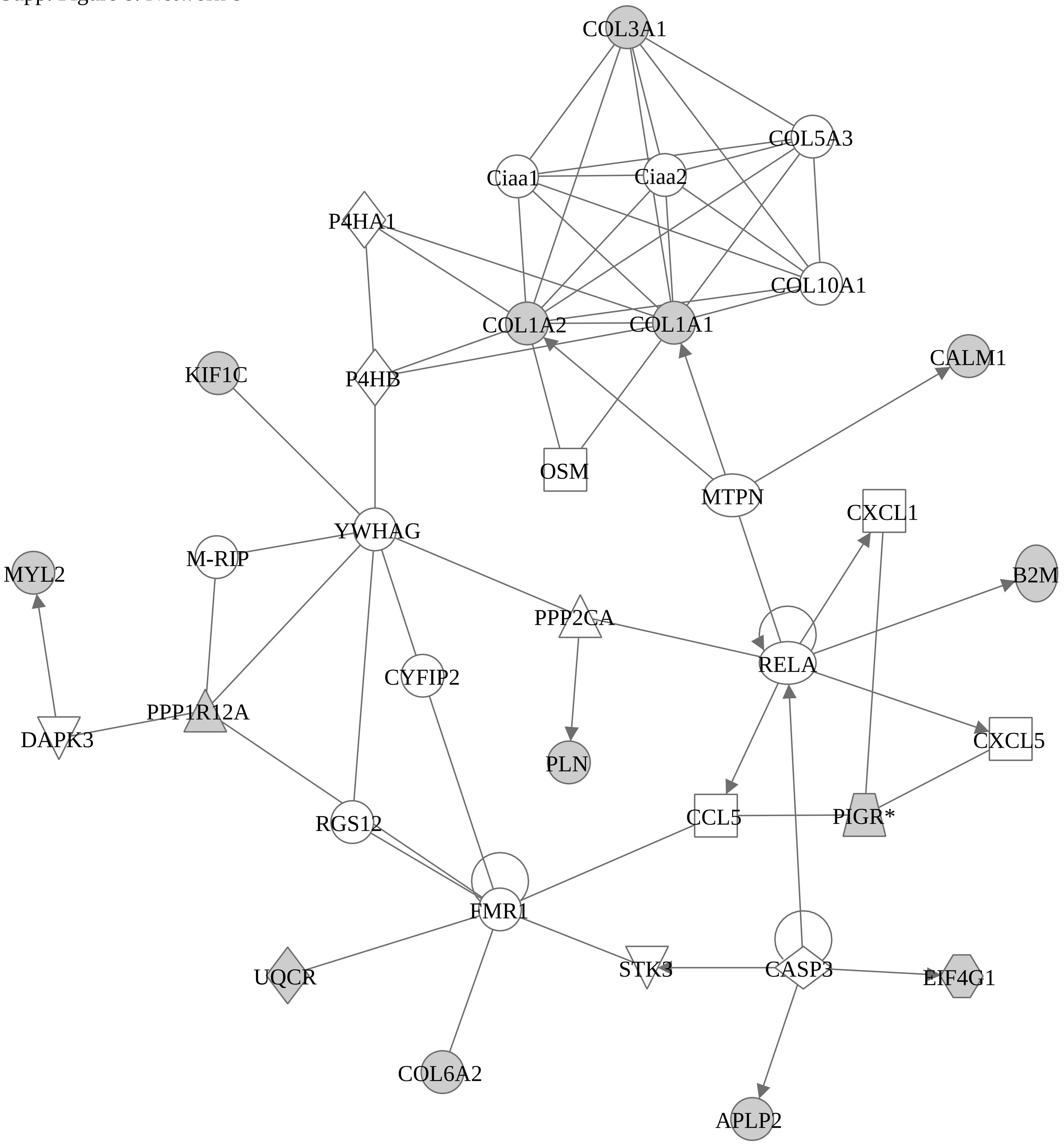

© 2000-2005, Ingenuity Systems

Supp. Figure 9. Network 9

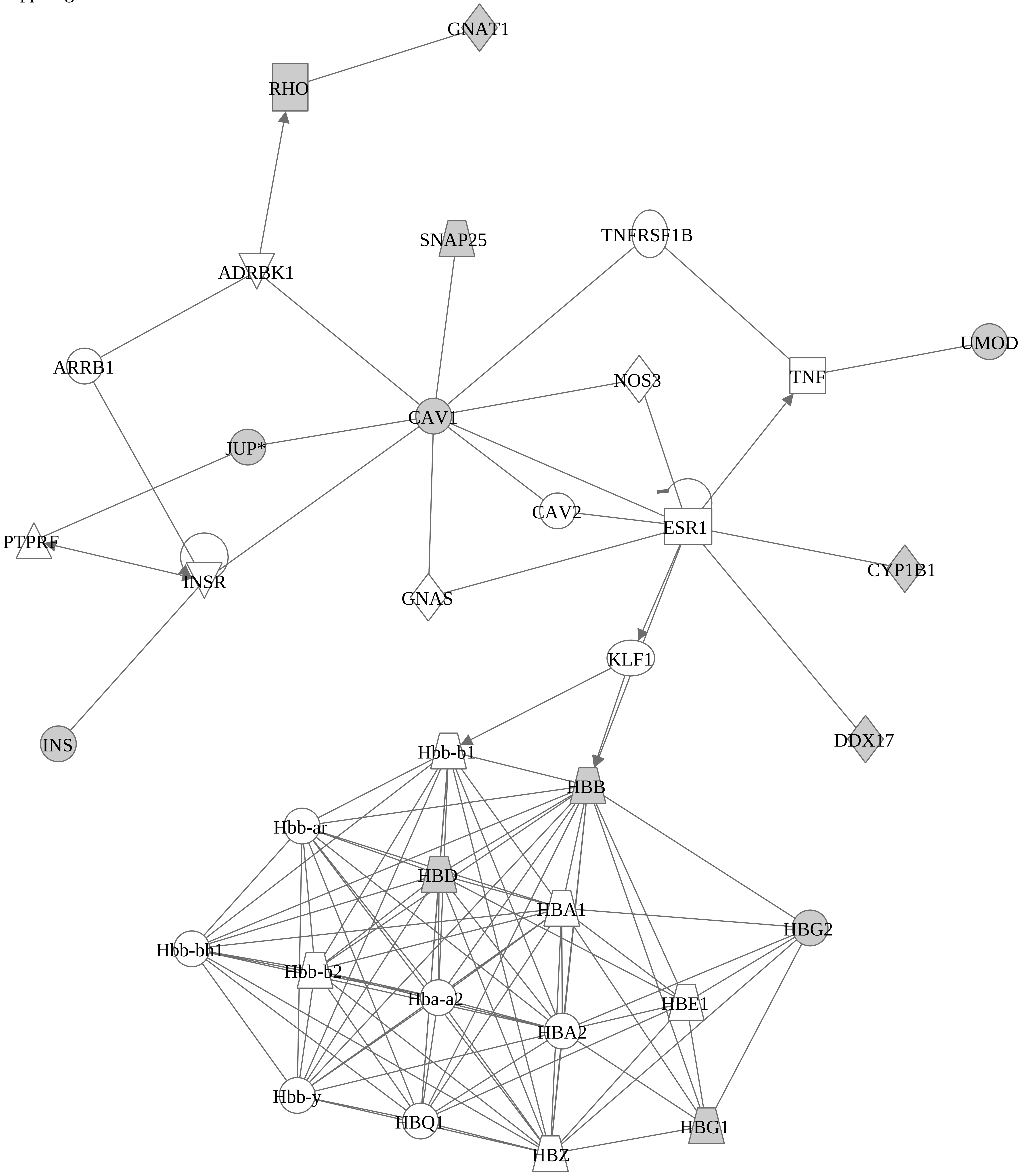

© 2000-2005, Ingenuity Systems

Supp. Figure 10. Network 10

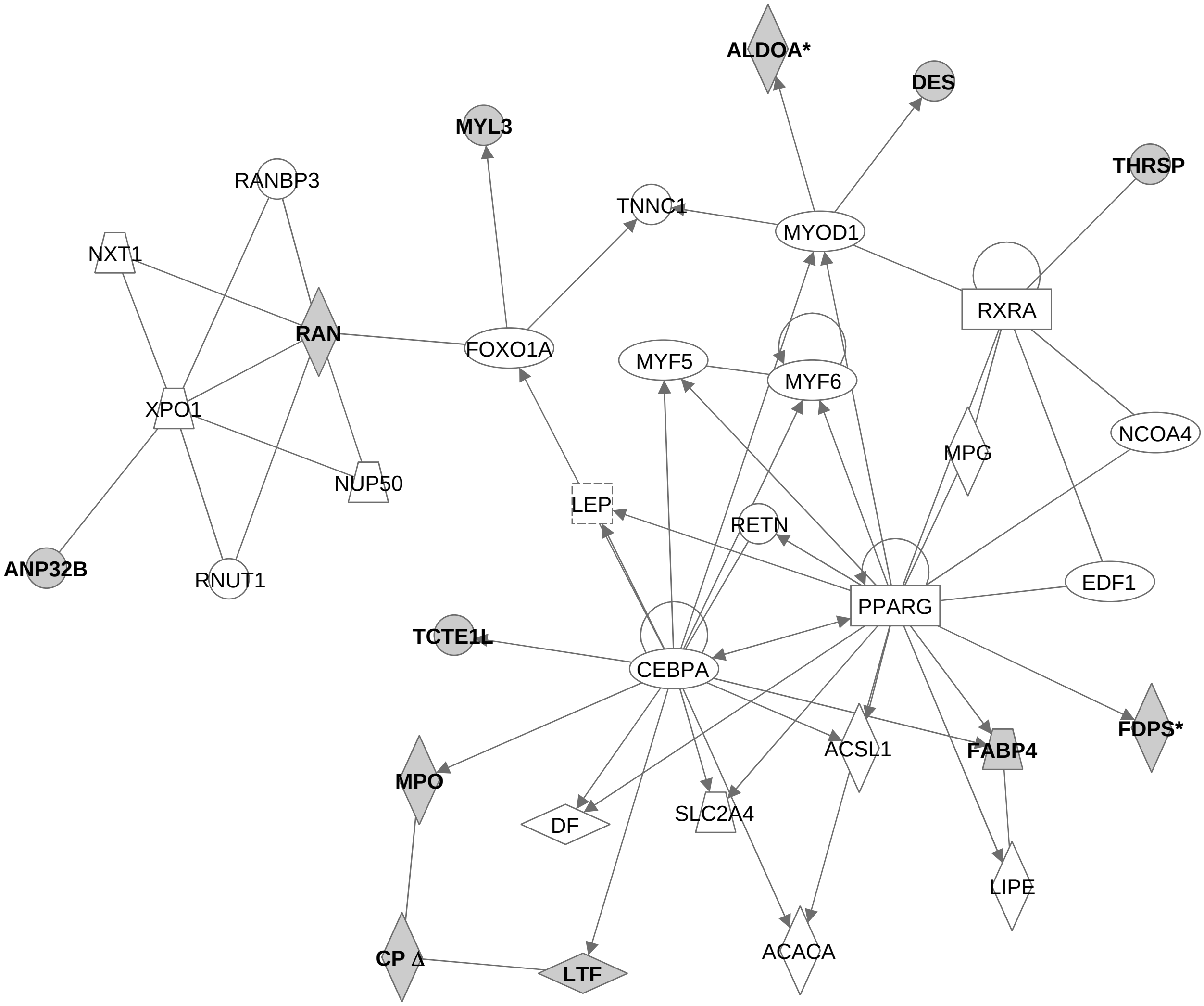

© 2000-2005, Ingenuity Systems
