# Supplementary material for: Mining expressed sequence tags identifies cancer markers of clinical interest
Source: BMC Bioinformatics. 2006 Nov 1;7:481. doi: 10.1186/1471-2105-7-481 (PMC1635568; doi:10.1186/1471-2105-7-481)
Supplement: Additional File 2 — Supplementary Table 1. Description and annotation of 39 genes identified by both the human and the mouse dbEST screens. [file 1471-2105-7-481-S2.doc]

**Supplementary Table 1. Identity and annotation of 39 genes found both by the human and mouse dbEST screens.**

| **Ensembl Gene ID** | **References discussing known dis-regulation in cancer** | **Gene Description** |
| --- | --- | --- |
| [ENSG00000008988](http://www.ensembl.org/Homo_sapiens/geneview?gene=ENSG00000008988) | [1] | 40S ribosomal protein S20. |
| [ENSG00000012223](http://www.ensembl.org/Homo_sapiens/geneview?gene=ENSG00000012223) | [2] | Lactotransferrin precursor (Lactoferrin) (Talalactoferrin alfa) |
| [ENSG00000019582](http://www.ensembl.org/Homo_sapiens/geneview?gene=ENSG00000019582) | [3] | HLA class II histocompatibility antigen gamma chain (HLA-DR antigens- associated invariant chain) (CD74 antigen). |
| [ENSG00000034510](http://www.ensembl.org/Homo_sapiens/geneview?gene=ENSG00000034510) | [4] | Thymosin beta-10. |
| [ENSG00000042493](http://www.ensembl.org/Homo_sapiens/geneview?gene=ENSG00000042493) | Member of the gelsolin family. | Macrophage capping protein (Actin-regulatory protein CAP-G). |
| [ENSG00000067225](http://www.ensembl.org/Homo_sapiens/geneview?gene=ENSG00000067225) |  | Pyruvate kinase isozymes M1/M2 (Cytosolic thyroid hormone-binding protein) (CTHBP) (THBP1). |
| [ENSG00000074800](http://www.ensembl.org/Homo_sapiens/geneview?gene=ENSG00000074800) | [5] | Alpha-enolase(2-phospho-D-glycerate hydro-lyase) (Non- neural enolase) (Enolase 1) (Phosphopyruvate hydratase) (C-myc promoter-binding protein) (Plasminogen-binding protein). |
| [ENSG00000090382](http://www.ensembl.org/Homo_sapiens/geneview?gene=ENSG00000090382) |  | Lysozyme C precursor (1,4-beta-N-acetylmuramidase C). |
| [ENSG00000091513](http://www.ensembl.org/Homo_sapiens/geneview?gene=ENSG00000091513) | [6,7] | Serotransferrin precursor (Transferrin) (Siderophilin) |
| [ENSG00000096384](http://www.ensembl.org/Homo_sapiens/geneview?gene=ENSG00000096384) | [8] | Heat shock protein HSP 90-beta (HSP 84) (HSP 90). |
| [ENSG00000108106](http://www.ensembl.org/Homo_sapiens/geneview?gene=ENSG00000108106) |  | Ubiquitin-conjugating enzyme E2S (Ubiquitin-conjugating enzyme E2-24 kDa) (Ubiquitin-protein ligase) |
| [ENSG00000108821](http://www.ensembl.org/Homo_sapiens/geneview?gene=ENSG00000108821) |  | Collagen alpha-1(I) chain precursor. |
| [ENSG00000109107](http://www.ensembl.org/Homo_sapiens/geneview?gene=ENSG00000109107) |  | Fructose-bisphosphate aldolase C (Brain-type aldolase). |
| [ENSG00000111057](http://www.ensembl.org/Homo_sapiens/geneview?gene=ENSG00000111057) | [9] | Keratin, type I cytoskeletal 18 (Cytokeratin-18) (CK-18) (Keratin-18) (K18). |
| [ENSG00000114867](http://www.ensembl.org/Homo_sapiens/geneview?gene=ENSG00000114867) | [10] | Eukaryotic translation initiation factor 4 gamma 1 (eIF-4-gamma 1) (eIF-4G1) (eIF-4G 1) (p220). |
| [ENSG00000115461](http://www.ensembl.org/Homo_sapiens/geneview?gene=ENSG00000115461) | [11] | Insulin-like growth factor-binding protein 5 precursor (IGFBP-5) (IBP- 5) (IGF-binding protein 5). |
| [ENSG00000117289](http://www.ensembl.org/Homo_sapiens/geneview?gene=ENSG00000117289) | [12] | thioredoxin interacting protein |
| [ENSG00000118271](http://www.ensembl.org/Homo_sapiens/geneview?gene=ENSG00000118271) |  | Transthyretin precursor (Prealbumin) (TBPA) (TTR) (ATTR). |
| [ENSG00000120885](http://www.ensembl.org/Homo_sapiens/geneview?gene=ENSG00000120885) | [13] | Clusterin precursor (Complement-associated protein SP-40,40) (Complement cytolysis inhibitor) (Apolipoprotein J) (Apo-J) (Testosterone-repressed prostate message 2) (TRPM-2) |
| [ENSG00000140988](http://www.ensembl.org/Homo_sapiens/geneview?gene=ENSG00000140988) |  | 40S ribosomal protein S2 (S4) (LLRep3 protein). |
| [ENSG00000149273](http://www.ensembl.org/Homo_sapiens/geneview?gene=ENSG00000149273) |  | 40S ribosomal protein S3. |
| [ENSG00000156508](http://www.ensembl.org/Homo_sapiens/geneview?gene=ENSG00000156508) |  | Elongation factor 1-alpha 1 (EF-1-alpha-1) (Elongation factor 1 A-1) (eEF1A-1) (Elongation factor Tu) (EF-Tu). |
| [ENSG00000162896](http://www.ensembl.org/Homo_sapiens/geneview?gene=ENSG00000162896) | [14] | Polymeric-immunoglobulin receptor precursor (Poly-Ig receptor) (PIGR) (Hepatocellular carcinoma-associated protein TB6) |
| [ENSG00000163914](http://www.ensembl.org/Homo_sapiens/geneview?gene=ENSG00000163914) |  | Rhodopsin (Opsin-2). |
| [ENSG00000167526](http://www.ensembl.org/Homo_sapiens/geneview?gene=ENSG00000167526) |  | 60S ribosomal protein L13 (Breast basic conserved protein 1). |
| [ENSG00000168542](http://www.ensembl.org/Homo_sapiens/geneview?gene=ENSG00000168542) |  | Collagen alpha-1(III) chain precursor. |
| [ENSG00000168925](http://www.ensembl.org/Homo_sapiens/geneview?gene=ENSG00000168925) | [15] | chymotrypsinogen B2 |
| [ENSG00000168928](http://www.ensembl.org/Homo_sapiens/geneview?gene=ENSG00000168928) | [15] | Chymotrypsinogen B precursor (EC 3.4.21.1) |
| [ENSG00000170421](http://www.ensembl.org/Homo_sapiens/geneview?gene=ENSG00000170421) | [16] | Keratin, type II cytoskeletal 8 (Cytokeratin-8) (CK-8) (Keratin-8) (K8). |
| [ENSG00000172757](http://www.ensembl.org/Homo_sapiens/geneview?gene=ENSG00000172757) | [17] | Cofilin-1 (Cofilin, non-muscle isoform) (18 kDa phosphoprotein) (p18). |
| [ENSG00000181163](http://www.ensembl.org/Homo_sapiens/geneview?gene=ENSG00000181163) | [18] | Nucleophosmin (NPM) (Nucleolar phosphoprotein B23) (Numatrin) (Nucleolar protein NO38). |
| [ENSG00000184009](http://www.ensembl.org/Homo_sapiens/geneview?gene=ENSG00000184009) | [19] | Actin, cytoplasmic 2 (Gamma-actin). |
| [ENSG00000186676](http://www.ensembl.org/Homo_sapiens/geneview?gene=ENSG00000186676) |  | Elongation factor 1-gamma (EF-1-gamma) (eEF-1B gamma). |
| [ENSG00000187021](http://www.ensembl.org/Homo_sapiens/geneview?gene=ENSG00000187021) |  | Pancreatic lipase-related protein 1 precursor (EC 3.1.1.3). |
| [ENSG00000188170](http://www.ensembl.org/Homo_sapiens/geneview?gene=ENSG00000188170) |  | Hemoglobin delta subunit (Hemoglobin delta chain) (Delta-globin). |
| [ENSG00000188229](http://www.ensembl.org/Homo_sapiens/geneview?gene=ENSG00000188229) |  | Tubulin beta-2C chain (Tubulin beta-2 chain). |
| [ENSG00000197249](http://www.ensembl.org/Homo_sapiens/geneview?gene=ENSG00000197249) | [20] | Alpha-1-antitrypsin precursor (Alpha-1 protease inhibitor) (Alpha-1- antiproteinase). |
| [ENSG00000197746](http://www.ensembl.org/Homo_sapiens/geneview?gene=ENSG00000197746) | [21] | Proactivator polypeptide precursor [Contains: Saposin A (Protein A); Saposin B-Val; Saposin B (Sphingolipid activator protein 1) (SAP-1) (Cerebroside sulfate activator) (CSAct) (Dispersin) (Sulfatide/GM1 activator) |
| [ENSG00000197971](http://www.ensembl.org/Homo_sapiens/geneview?gene=ENSG00000197971) | [22] | Myelin basic protein (MBP) (Myelin A1 protein) (Myelin membrane encephalitogenic protein). |

**References**

1. Karan D, Kelly DL, Rizzino A, Lin M, Batra SK (2002) Expression profile of differentially-regulated genes during progression of androgen-independent growth in human prostate cancer cells. Carcinogenesis 23: 967-975.

2. Benaissa M, Peyrat J, Hornez L, Mariller C, Mazurier J, et al. (2005) Expression and prognostic value of lactoferrin mRNA isoforms in human breast cancer. Int J Cancer 114: 299-306.

3. Ransom JH, Pelle B, Hanna MG (1992) Expression of class II major histocompatibility complex molecules correlates with human colon tumor vaccine efficacy. Cancer Res 52: 3460-3466.

4. Maxwell PJ, Longley DB, Latif T, Boyer J, Allen W, et al. (2003) Identification of 5-fluorouracil-inducible target genes using cDNA microarray profiling. Cancer Res 63: 4602-4606.

5. Ghosh AK, Steele R, Ray RB (2005) c-myc Promoter-binding protein 1 (MBP-1) regulates prostate cancer cell growth by inhibiting MAPK pathway. J Biol Chem 280: 14325-14330.

6. Ahmed N, Oliva KT, Barker G, Hoffmann P, Reeve S, et al. (2005) Proteomic tracking of serum protein isoforms as screening biomarkers of ovarian cancer. Proteomics 5: 4625-4636.

7. Ryschich E, Huszty G, Knaebel HP, Hartel M, Buchler MW, et al. (2004) Transferrin receptor is a marker of malignant phenotype in human pancreatic cancer and in neuroendocrine carcinoma of the pancreas. Eur J Cancer 40: 1418-1422.

8. Hoos A, Levey DL (2003) Vaccination with heat shock protein-peptide complexes: from basic science to clinical applications. Expert Rev Vaccines 2: 369-379.

9. Schaller G, Fuchs I, Pritze W, Ebert A, Herbst H, et al. (1996) Elevated keratin 18 protein expression indicates a favorable prognosis in patients with breast cancer. Clin Cancer Res 2: 1879-1885.

10. Tomonaga T, Matsushita K, Yamaguchi S, Oh-Ishi M, Kodera Y, et al. (2004) Identification of altered protein expression and post-translational modifications in primary colorectal cancer by using agarose two-dimensional gel electrophoresis. Clin Cancer Res 10: 2007-2014.

11. Butt AJ, Dickson KA, McDougall F, Baxter RC (2003) Insulin-like growth factor-binding protein-5 inhibits the growth of human breast cancer cells in vitro and in vivo. J Biol Chem 278: 29676-29685.

12. Steeg PS, Ouatas T, Halverson D, Palmieri D, Salerno M (2003) Metastasis suppressor genes: basic biology and potential clinical use. Clin Breast Cancer 4: 51-62.

13. Shannan B, Seifert M, Leskov K, Willis J, Boothman D, et al. (2006) Challenge and promise: roles for clusterin in pathogenesis, progression and therapy of cancer. Cell Death Differ 13: 12-19.

14. Bruno MEC, West RB, Schneeman TA, Bresnick EH, Kaetzel CS (2004) Upstream stimulatory factor but not c-Myc enhances transcription of the human polymeric immunoglobulin receptor gene. Mol Immunol 40: 695-708.

15. Kuroki T, Tomioka T, Tajima Y, Inoue K, Ikematsu Y, et al. (1999) Detection of the pancreas-specific gene in the peripheral blood of patients with pancreatic carcinoma. Br J Cancer 81: 350-353.

16. Hembrough TA, Li L, Gonias SL (1996) Cell-surface cytokeratin 8 is the major plasminogen receptor on breast cancer cells and is required for the accelerated activation of cell-associated plasminogen by tissue-type plasminogen activator. J Biol Chem 271: 25684-25691.

17. Wang W, Mouneimne G, Sidani M, Wyckoff J, Chen X, et al. (2006) The activity status of cofilin is directly related to invasion, intravasation, and metastasis of mammary tumors. J Cell Biol 173: 395-404.

18. Tsui K, Cheng A, Chang P, Pan T, Yung BY (2004) Association of nucleophosmin/B23 mRNA expression with clinical outcome in patients with bladder carcinoma. Urology 64: 839-844.

19. Suzuki H, Nagata H, Shimada Y, Konno A (1998) Decrease in gamma-actin expression, disruption of actin microfilaments and alterations in cell adhesion systems associated with acquisition of metastatic capacity in human salivary gland adenocarcinoma cell clones. Int J Oncol 12: 1079-1084.

20. Wojtukiewicz MZ, Rucinska M, Kloczko J, Dib A, Galar M (1998) Profiles of plasma serpins in patients with advanced malignant melanoma, gastric cancer and breast cancer. Haemostasis 28: 7-13.

21. Nagano H, Noguchi T, Inagaki K, Yoon S, Matozaki T, et al. (2003) Downregulation of stomach cancer-associated protein tyrosine phosphatase-1 (SAP-1) in advanced human hepatocellular carcinoma. Oncogene 22: 4656-4663.

22. Thomson DM, Springer GF, Desai PR, Scanzano R, Gubersky M, et al. (1988) Comparison by leukocyte adherence inhibition of human immune response to cancer-associated immunogens, Thomsen-Friedenreich (T) and Tn, myelin basic protein, and organ-specific cancer neoantigens. Clin Immunol Immunopathol 49: 231-241.
