# Supplementary material for: Mining expressed sequence tags identifies cancer markers of clinical interest
Source: BMC Bioinformatics. 2006 Nov 1;7:481. doi: 10.1186/1471-2105-7-481 (PMC1635568; doi:10.1186/1471-2105-7-481)
Supplement: Additional file 6 — Supplementary Table 5. Description of the gene lists. [file 1471-2105-7-481-S6.doc]

**Supplementary Table 5. Gene lists used in the microarray evaluation study.**

| **Name** | **Number of unique genes** | **Reference** |
| --- | --- | --- |
| GDS1070 | 82 | [1] |
| GDS183 | 28 | [2] |
| GDS232 | 85 | [3] |
| NuttAO | 76 | [4] |
| NuttGBM | 50 | [4] |
| Pomeroy-400 | 305 | [5] |
| Pomeroy-0 | 166 | [5] |
| Pomeroy-1 | 102 | [5] |
| Pomeroy-2 | 132 | [5] |
| Pomeroy-3 | 95 | [5] |
| Pomeroy-4 | 92 | [5] |
| Rhodes2004 | 57 | [6] |
| Perou-Brown-Botstein1999 | 31 | [7] |
| van’tVeer | 92 | [8] |
| HM200 | 200 | this paper |
| HM100 | 100 | this paper |
| HM50 | 50 | this paper |
| HM10 | 10 | this paper |
| NC01-2000 | 420 | this paper |
| full | depends on study | see Supp. Table 2 |

Many of these gene lists were selected in different studies as being transcriptional cancer biomarkers (i.e., genes whose expression levels correlate with patient classes of clinical interest). NC01-2000 is a list of genes not expected to be differentially expressed between classes (negative control). The full gene list represents the total number of probesets on the array used in each study (positive control). The number of unique genes in each list and the primary reference are listed.

**References**

1. Cromer A, Carles A, Millon R, Ganguli G, Chalmel F, et al. (2004) Identification of genes associated with tumorigenesis and metastatic potential of hypopharyngeal cancer by microarray analysis. Oncogene 23: 2484-2498.

2. Dyrskjot L, Thykjaer T, Kruhoffer M, Jensen JL, Marcussen N, et al. (2003) Identifying distinct classes of bladder carcinoma using microarrays. Nat Genet 33: 90-96.

3. MacDonald TJ, Brown KM, LaFleur B, Peterson K, Lawlor C, et al. (2001) Expression profiling of medulloblastoma: PDGFRA and the RAS/MAPK pathway as therapeutic targets for metastatic disease. Nat Genet 29: 143-152.

4. Nutt CL, Mani DR, Betensky RA, Tamayo P, Cairncross JG, et al. (2003) Gene expression-based classification of malignant gliomas correlates better with survival than histological classification. Cancer Res 63: 1602-1607.

5. Pomeroy SL, Tamayo P, Gaasenbeek M, Sturla LM, Angelo M, et al. (2002) Prediction of central nervous system embryonal tumour outcome based on gene expression. Nature 415: 436-442.

6. Rhodes DR, Yu J, Shanker K, Deshpande N, Varambally R, et al. (2004) Large-scale meta-analysis of cancer microarray data identifies common transcriptional profiles of neoplastic transformation and progression. Proc Natl Acad Sci U S A 101: 9309-9314.

7. Perou CM, Jeffrey SS, van de Rijn M, Rees CA, Eisen MB, et al. (1999) Distinctive gene expression patterns in human mammary epithelial cells and breast cancers. Proc Natl Acad Sci U S A 96: 9212-9217.

8. van 't Veer LJ, Dai H, van de Vijver MJ, He YD, Hart AA, et al. (2002) Gene expression profiling predicts clinical outcome of breast cancer. Nature 415: 530-536.
